# Supplementary material for: Detection of ferrihydrite in Martian red dust records ancient cold and wet conditions on Mars
Source: Nat Commun. 2025 Feb 25;16:1712. doi: 10.1038/s41467-025-56970-z (PMC11861699; doi:10.1038/s41467-025-56970-z)
Supplement: Supplementary file 1 — Supplementary Information [file 41467_2025_56970_MOESM1_ESM.pdf]

Detection of ferrihydrite in Martian red dust records ancient cold and wet conditions on Mars

Supplementary information file

Band depth and slope analysis

Close inspection of the spectral slope (S600, S750) and band depth (BD535, BD600) parameters[1] for ChemCam, OMEGA, CRISM, ferrihydrite-basalt, and hematite-basalt spectra (Supplementary Fig. 8) indicates that in the visible wavelengths (500–850 nm), the ferrihydrite-basalt mixture provides a better spectral match to the observations of Martian dust. The selection of wavelengths for band depth and slope analysis depends on the specific type of feature that is being analyzed. For example, the BD600 parameter is highly sensitive to the aforementioned spectral shoulder due to the $^6A_1 \rightarrow ^4T_2$ transition. This explains the observed offset between the ferrihydrite-basalt mix and Martian observation data points.

Dehydration experiment

The electron transition $^6A_1 \rightarrow ^4T_1$ band of ferrihydrite (at ~0.95 μm), along with its left wing, exhibited a noticeable loss in contrast following dehydration in agreement with a recent study (see their Fig. 6b)[2]. We highlight the change of this band by the reflectance ratio before and after the experiment (Supplementary Fig. 12). This phenomenon suggests that the removal of water and/or hydroxyl groups results in further deformation of the crystal structure by disrupting the connections between adjacent Fe^{3+} atoms and intermediate OH/H_2O molecules. These observations are consistent with previous dehydration experiments, which demonstrated that

some ferric-bearing oxyhydroxides lose contrast at the $^{6}A_1 \rightarrow ^{4}T_1$ band as a result of losing Fe^{3+}-bridging hydroxyls[3,4]. This may explain the presence of a very weak Fe band (at ~1.0 µm, Figure 1c main text), in the Martian dust spectrum, which is also shifted to slightly longer wavelengths. This observation is consistent with our ferrihydrite-basalt mixture spectra, as evidenced by the major electronic transition band minima position measurements (Supplementary Fig. 3). The dehydration of ferrihydrite-basalt mixture also reveals changes and loss of contrast in the range 550 nm to the reflectance maximum at ~800 nm. This suggests that the alterations observed in the electronic transition band are structurally insignificant, as the XRD data do not reveal significant changes in crystallinity.

Ferrihydrite stability estimations

In aqueous solutions

The transformation rate of ferrihydrite to hematite was calculated following ref.[5]. The Arrhenius equation was used to determine the rate constant (k) for ferrihydrite transformation at low temperatures:

$$k = A \cdot e^{\frac{-E_a}{RT}}$$

where: k is the reaction rate constant (h^-1). A is the pre-exponential factor (1.14 × 10^12 h^-1), Ea is the activation energy (97,259 J/mol), R is the universal gas constant (8.314 J/mol·K), T is temperature in Kelvin. Using the experimentally determined activation energy and pre-exponential factor from ref.[6] we extrapolated the rate constant to environmentally relevant conditions (pH 2, 1°C). The resulting rate constant was used in the first-order reaction equation:

$$[A]_t = [A]_0 \cdot e^{-kt}$$

where [A]*t* is the amount of ferrihydrite remaining at time *t*, [A]0 is the initial amount, *k* is the rate constant, and *t* is time. Under these conditions (pH 2, 1°C), calculations indicate approximately 1000 years would be required for complete transformation of ferrihydrite to hematite.

Supplementary Table

Supplementary Table 1. Mixing ratios used in this study.

Sample	Ratio
Ferrihydrite-basalt 1	1:99
Ferrihydrite-basalt 2	1:9
Ferrihydrite-basalt 3	1:3
Ferrihydrite-basalt 4	1:2
Ferrihydrite-basalt 5	1:1
Hematite-basalt 1	1:99
Hematite-basalt 2	1:9
Hematite-basalt 3	1:2
Hematite-basalt 4	1:1
Ferrihydrite-basalt-goethite 1	3:6:1
Ferrihydrite-basalt-goethite 2	33:66:1
Ferrihydrite-basalt-hematite 1	3:6:1
Ferrihydrite-basalt-hematite 2	33:66:1
Ferrihydrite-basalt-Mg sulfate 1	1:2:1
Ferrihydrite-basalt-Mg sulfate 2	2:4:1
Schwertmannite-basalt	1:2
Akaganeite-basalt	1:2
Goethite-basalt	1:2
Schwertmannite-basalt	1:2
Akaganeite-basalt	1:2
Goethite-basalt	1:2

Supplementary Figures

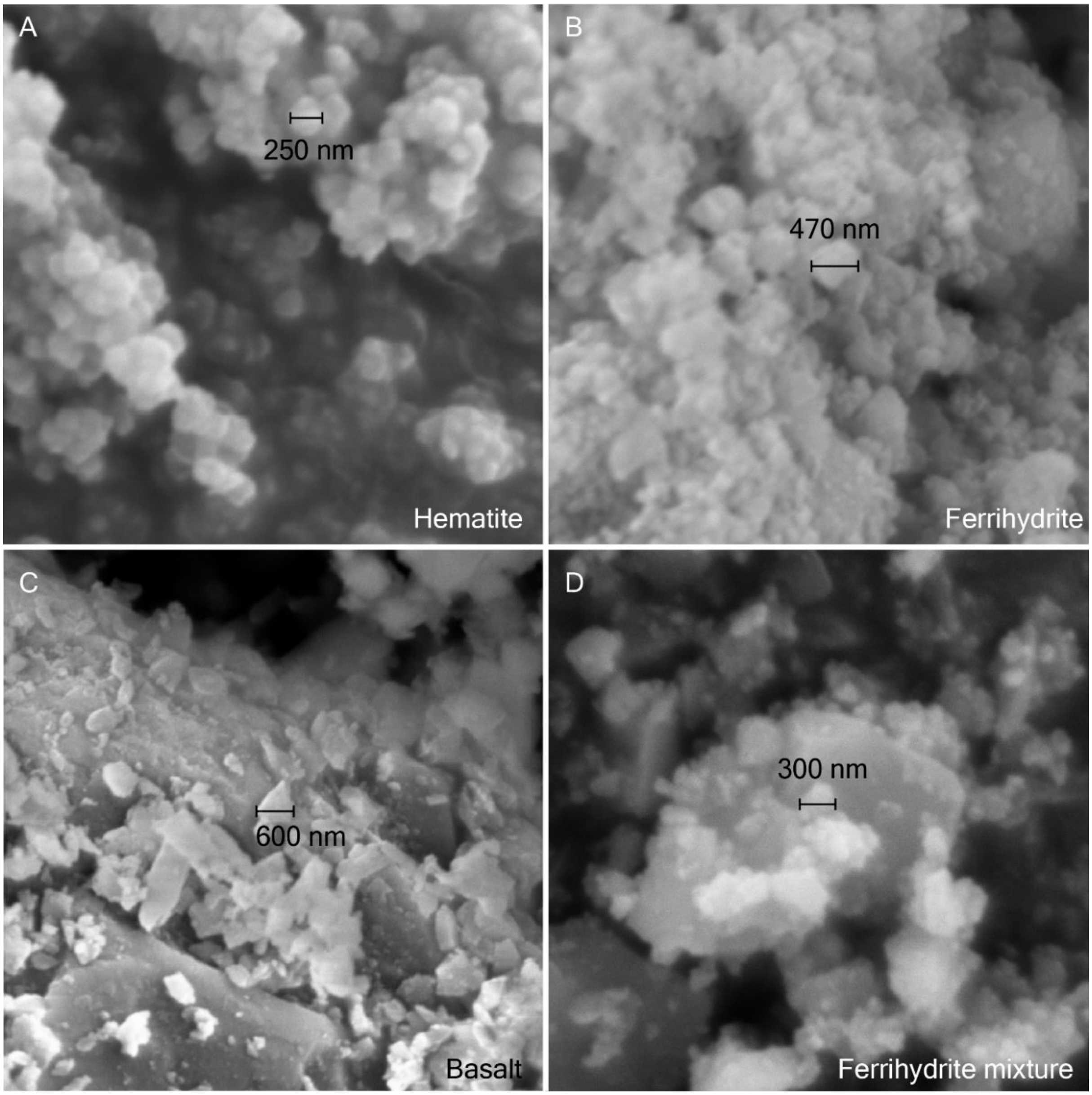

Supplementary Fig. 1. Scanning Electron Microscope (SEM) images of samples used in this work. (a) Industrial nanophase hematite powder acquired from Sigma Aldrich, (b,c) lab ground hyperfine ferrihydrite and basalt, and (d) ferrihydrite-basalt mixture. In all cases the particle size

is below 1 micron. Note that the particles observed here of the synthetic ferrihydrite are likely agglomerates since its crystallites are on the order of <10 nm in size[7].

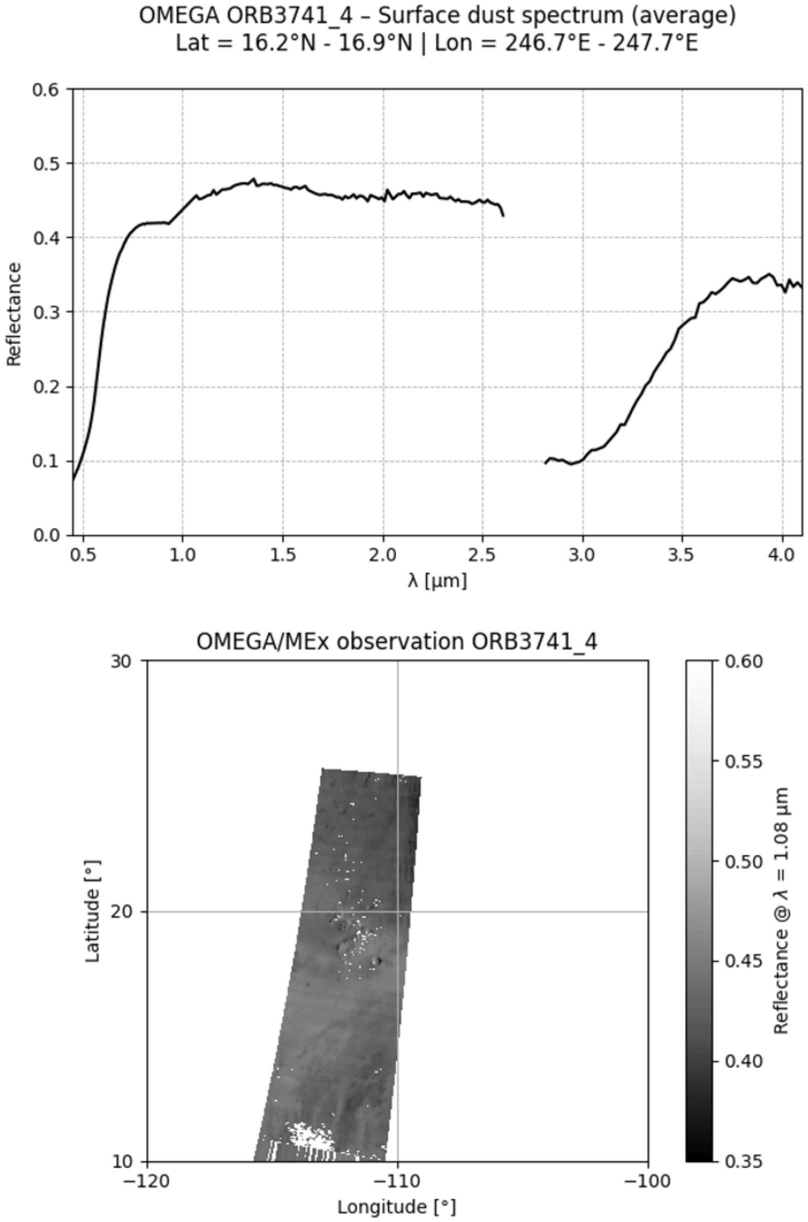

Supplementary Fig. 2. OMEGA observation of spectrally bland and dusty region in Tharsis. The sudden jump in reflectance at about 1 micron is due to detector cutoff.

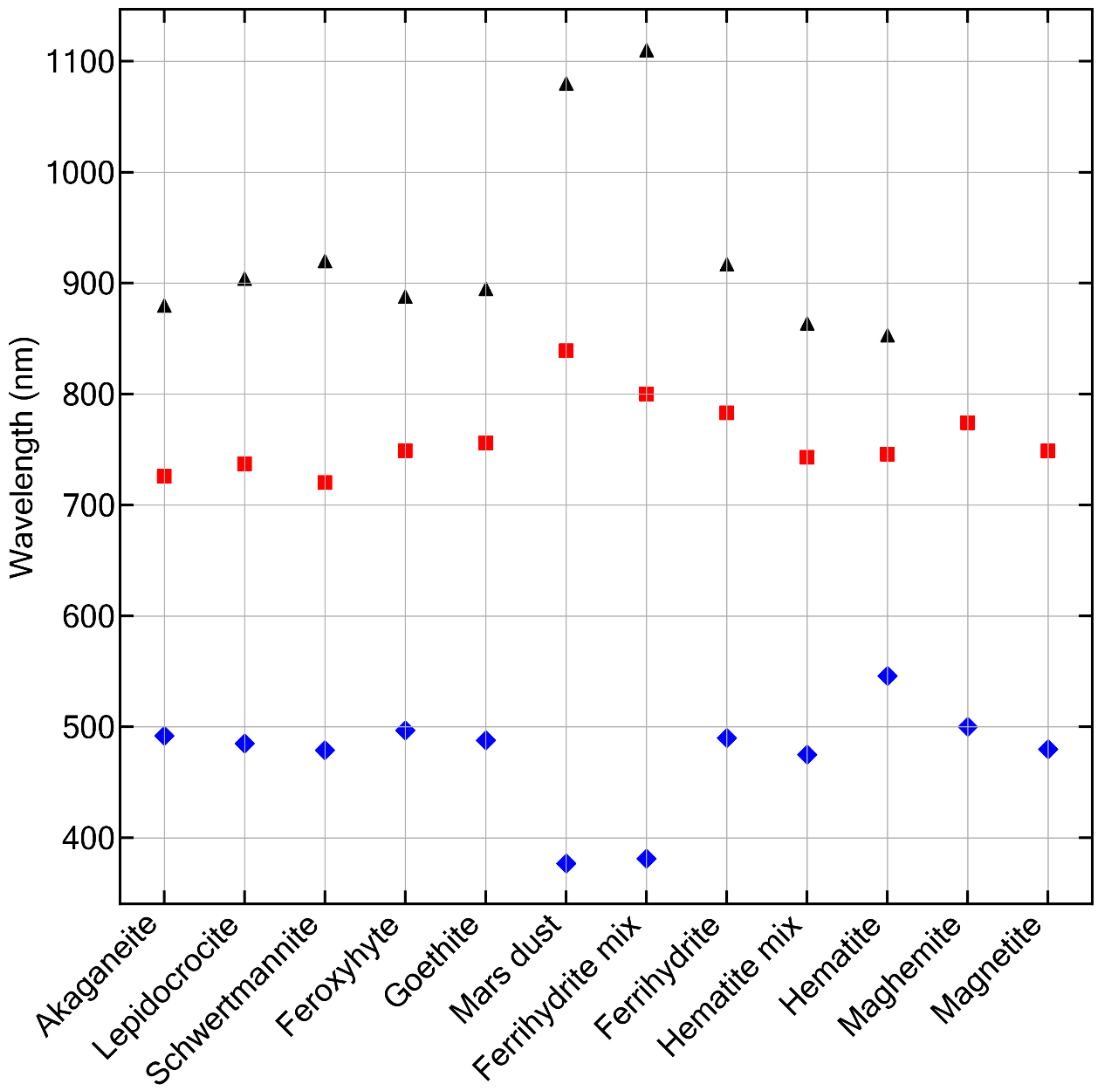

Supplementary Fig. 3. Major electronic transition band minima positions for pure iron (oxy)hydroxides, their mixtures, and Martian dust. Determined from the samples and data used in this study.

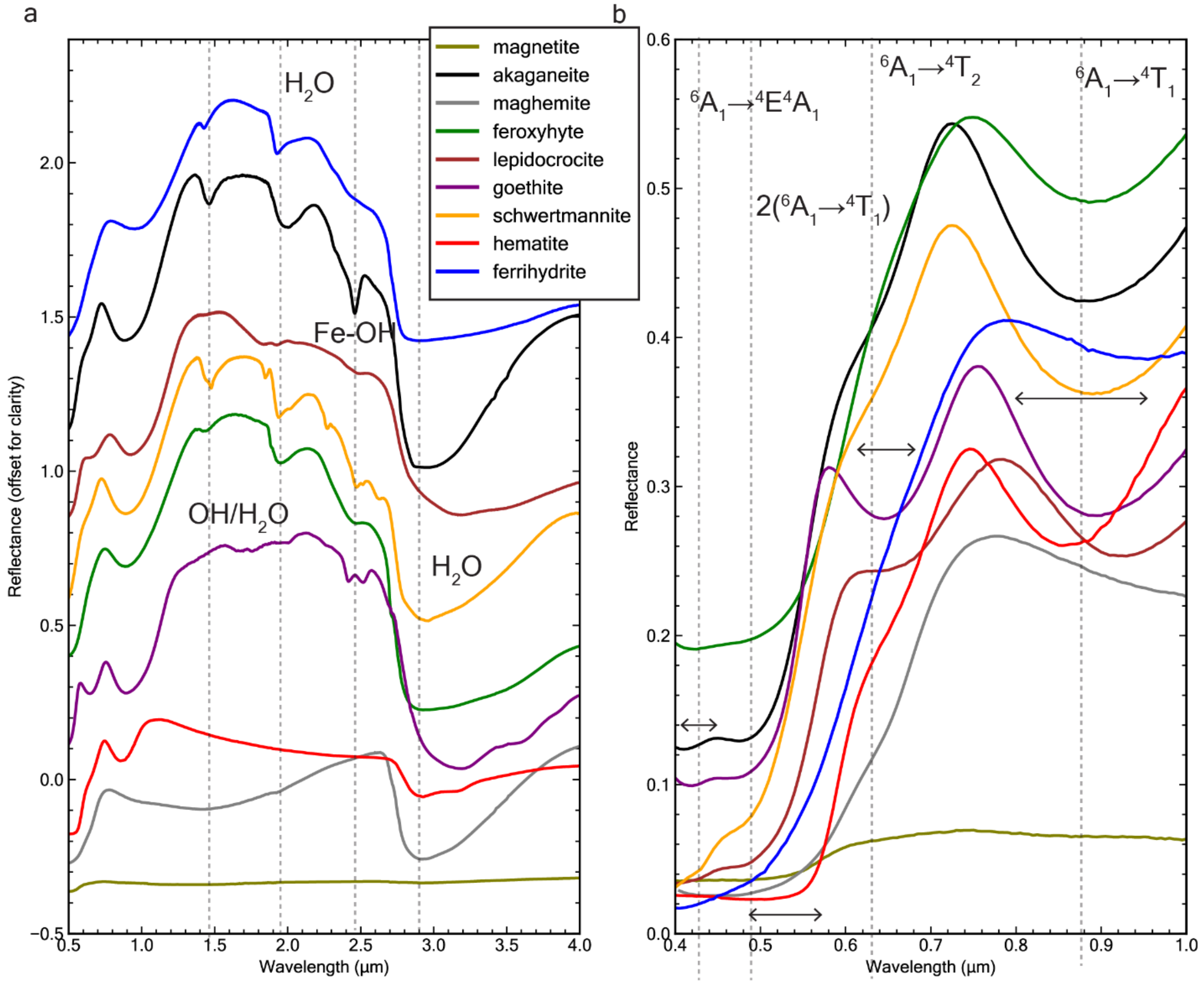

Supplementary Fig. 4. Ferrihydrite's VNIR spectrum compared to other iron oxides and (oxy)hydroxides. (a) Stacked spectra showing major OH/H_2O stretching/bending vibrational bands. (b) Close up of the characteristic visible to near-infrared region (0.4-1.0 µm) where major electronic transitions occur in iron oxides/oxy(hydroxides), leading to absorption features. Note the shallow ${}^6A_1 \rightarrow {}^4T_1$ band of ferrihydrite at about 0.95 µm in contrast to the very deep band in all other oxide spectra. This observation is in agreement with multiple laboratory studies[8,9]. The ferrihydrite spectrum also does not exhibit the ${}^6A_1 \rightarrow {}^4T_2$ transition feature. All spectra acquired under ambient conditions. Particle sizes: ferrihydrite and hematite (<1 µm), goethite (<11 µm),

lepidocrocite (<40 μm), akaganeite (< 30 μm), maghemeite (<20 μm), schwertmannite (<30 μm), feroxyhyte (<40 μm), and magnetite (<10 μm). Note that XRD analysis revealed minor goethite impurities in the lepidocrocite sample and natrojarosite in the schwertmannite sample. Source data are provided as a Source Data file.

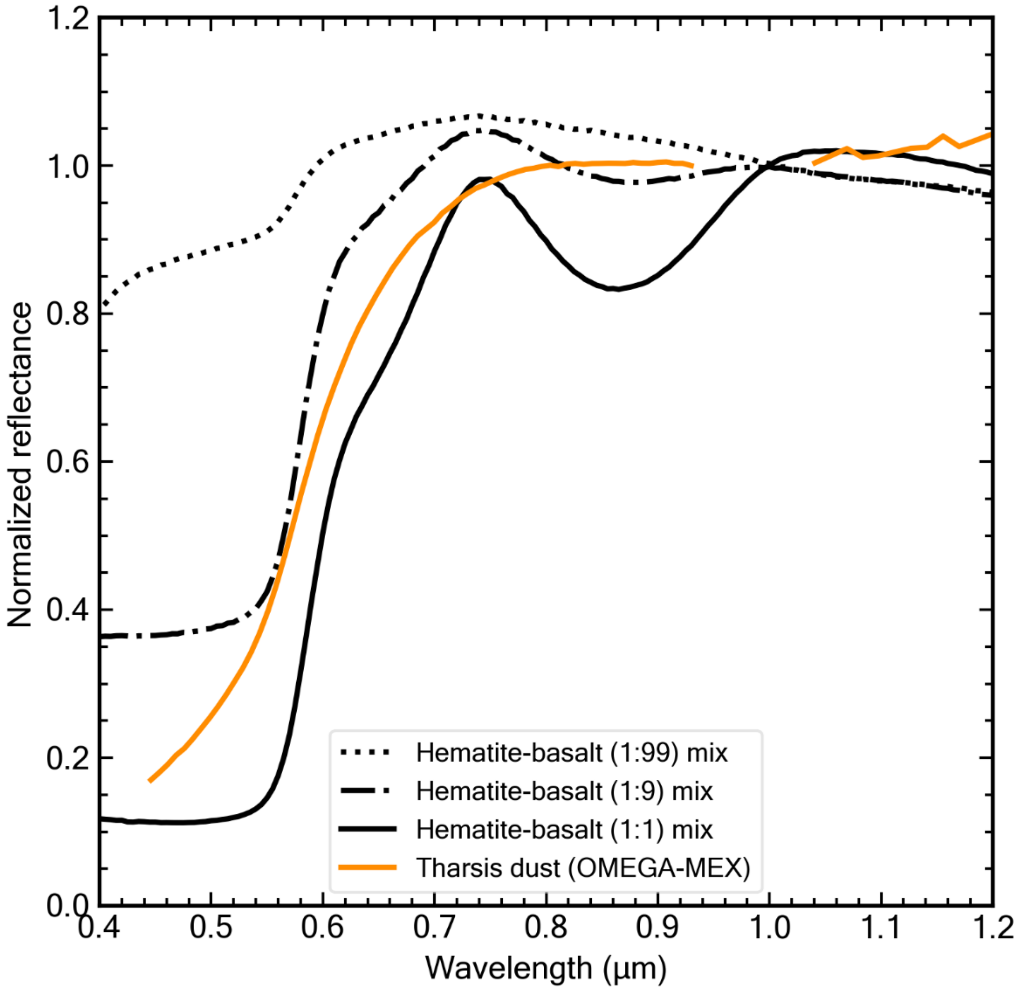

Supplementary Fig. 5. Hematite-basalt mix spectra of different weight ratios in comparison OMEGA spectrum of Martian dust. The 1:99 weight ratio produces a spectrum distinctly different from that of Martian dust, while a 1:9 ratio exhibits clear hematite spectral signatures

(broad blue absorption at ~0.5 μm, shoulder at 0.6 μm and deep absorption at 0.85 μm) in the VNIR range that are not observed in Martian spectra. These results strongly suggest that hematite cannot be the dominant iron oxide phase in Martian dust. Spectra normalized at 1.0 μm. Source data are provided as a Source Data file.

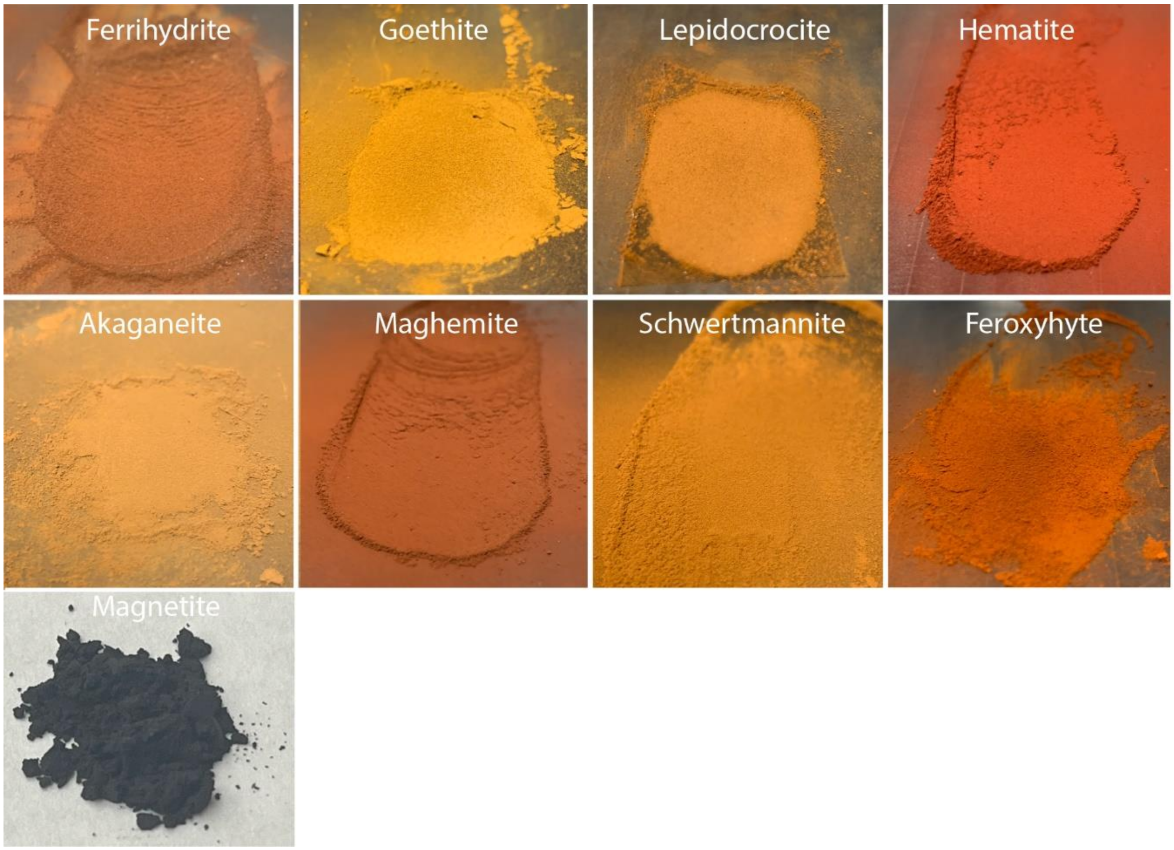

Supplementary Fig. 6. The different colors of iron oxides and (oxy)hydroxides investigated in this work. The samples were compressed and sprinkled with the remaining powder using a very fine nylon mesh. The sprinkling procedure was not effective for all samples due to powder agglomeration in some cases.

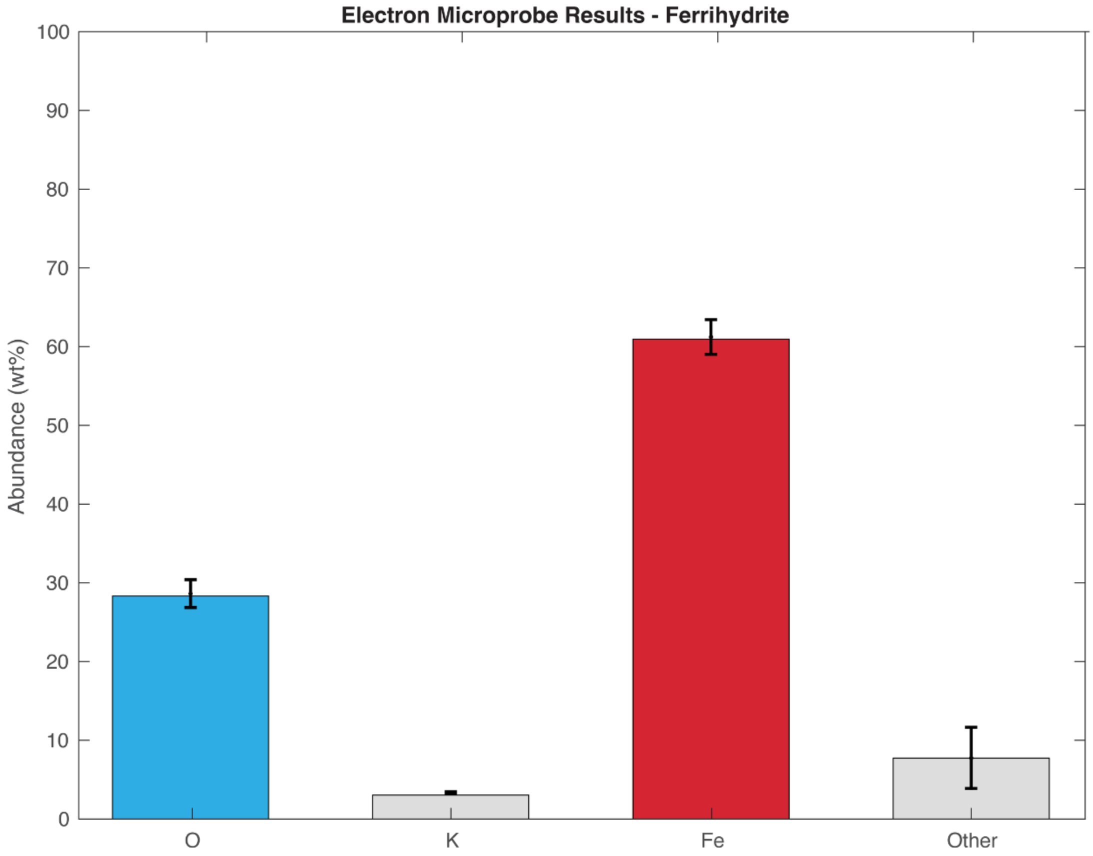

Supplementary Fig. 7. Elemental composition of synthetic ferrihydrite by electron microprobe analysis. The mass deficit, which is categorized as 'Other' likely comprises sodium or similar impurities and may reflect surface degradation from electron beam exposure - a common phenomenon in hydrated mineral analysis. Trace potassium contamination was attributed to the synthesis protocol detailed in Methods.

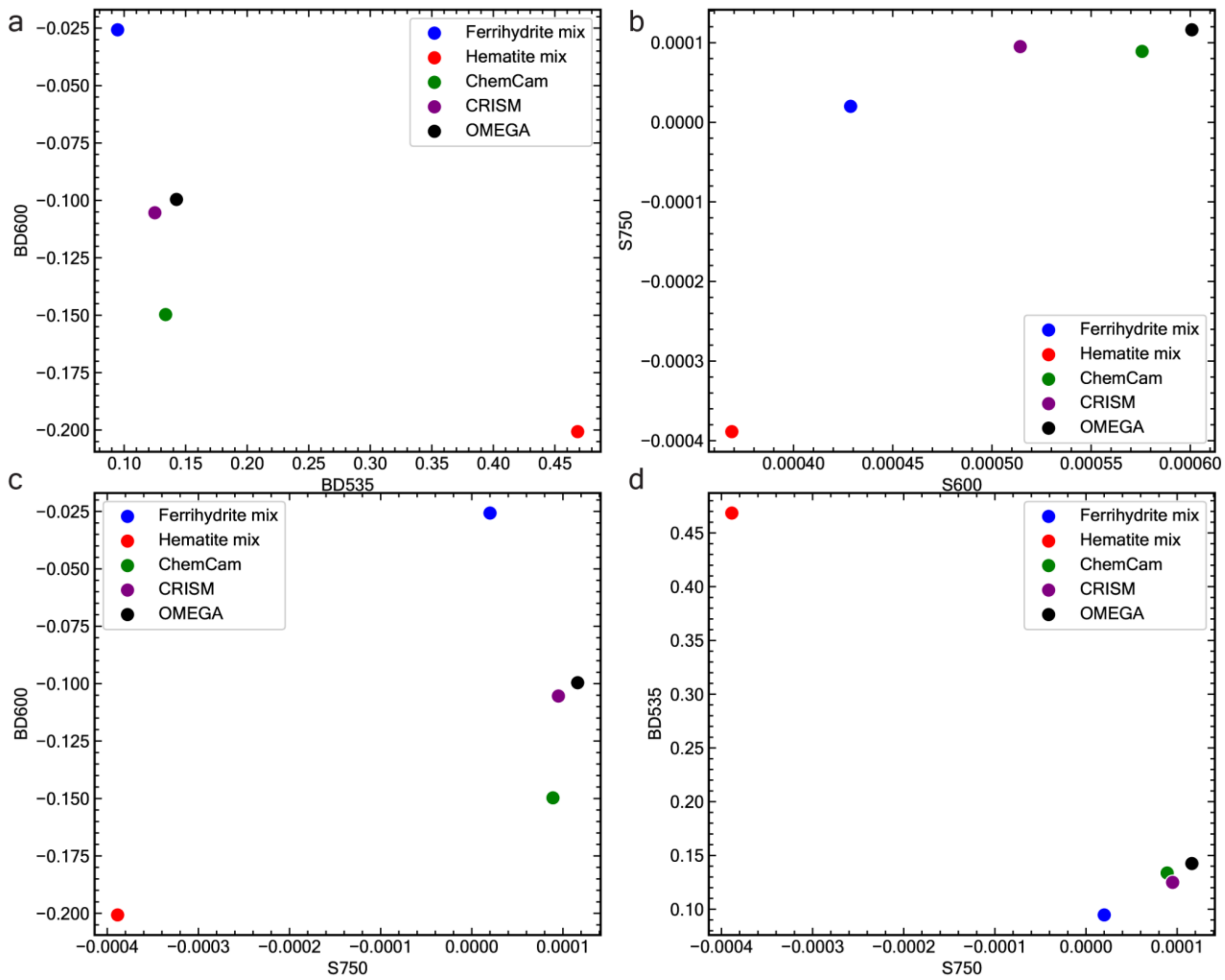

Supplementary Fig. 8. Spectral parameter plots for ferrihydrite, hematite, and Martian spectra. (a) Comparison of band depth BD535 vs BD600, (b) slope S600 vs S750, (c) S750 vs BD600, and (d) S750 vs BD535 spectral parameters. The spectral parameter analysis indicates that Martian dust, in the range of approximately 500 nm to 840 nm, more closely resembles the ferrihydrite-basalt mixture than the hematite-basalt spectrum. The spectral slope and band depth parameters were defined according to the criteria established by ref.[1]. The spectral shoulder at ~600 nm is highlighted by the BD600 parameter in (a) and (c).

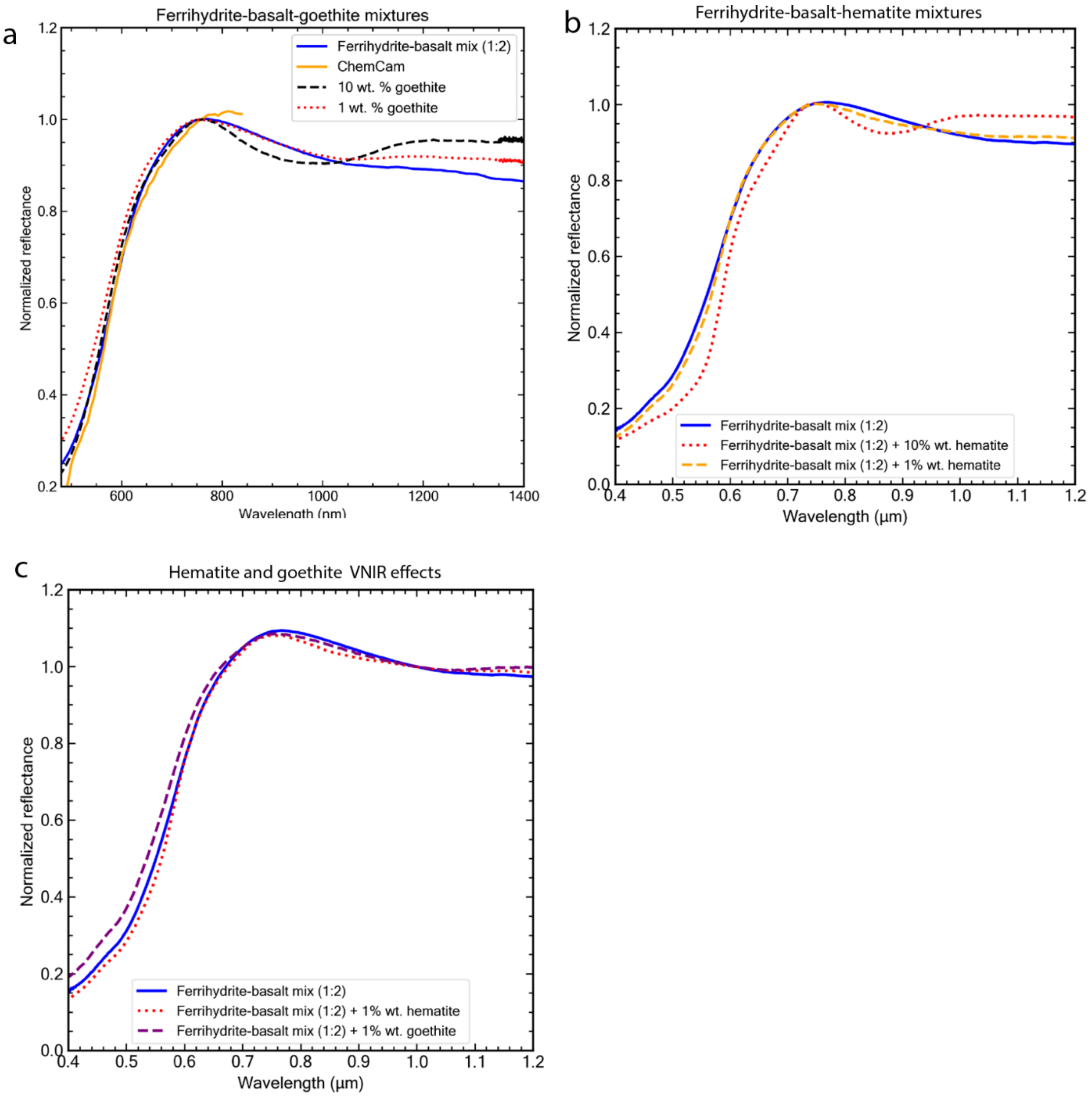

Supplementary Fig. 9. VNIR spectra of ferrihydrite-basalt mixtures with (a) goethite, (b) hematite, and (c) comparison of goethite and hematite additions. The observed weak shoulder at 600 nm in the Martian dust spectrum may be due to very small amounts of (oxy)hydroxides in addition to ferrihydrite. Both 1 wt% goethite and 1 wt% hematite may provide better fits with the ferrihydrite-basalt mixture spectrum, though caution is warranted as spectral differences at such

low concentrations are subtle and somewhat ambiguous. Higher concentrations (10 wt%) result in deeper Fe bands (at 0.9 µm for goethite and 0.86 µm for hematite) that are not observed on Mars. All spectra normalized at 740 nm.

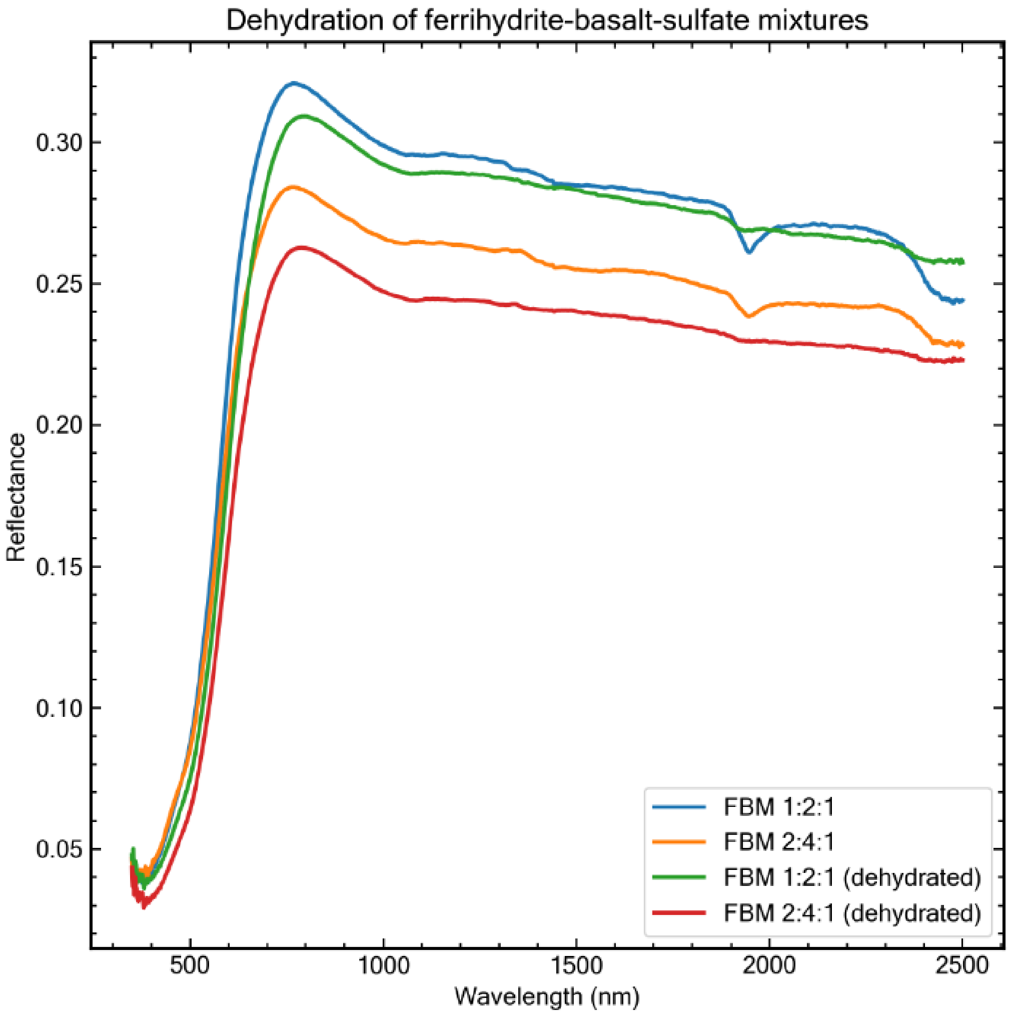

Supplementary Fig. 10. Dehydration experiment of ferrihydrite-basalt-Mg-sulfate mixtures. Reflectance spectra of ferrihydrite-basalt-Mg-sulfate mixtures in two different ratios, before and

after a 40-day dehydration experiment. The notable 1.4-, 1.9- and 2.4-µm hydration features are not observed after dehydration. The dehydrated spectra acquired at the University of Winnipeg, do not include the 3-micron band due to spectral range limitations of the spectrometer.

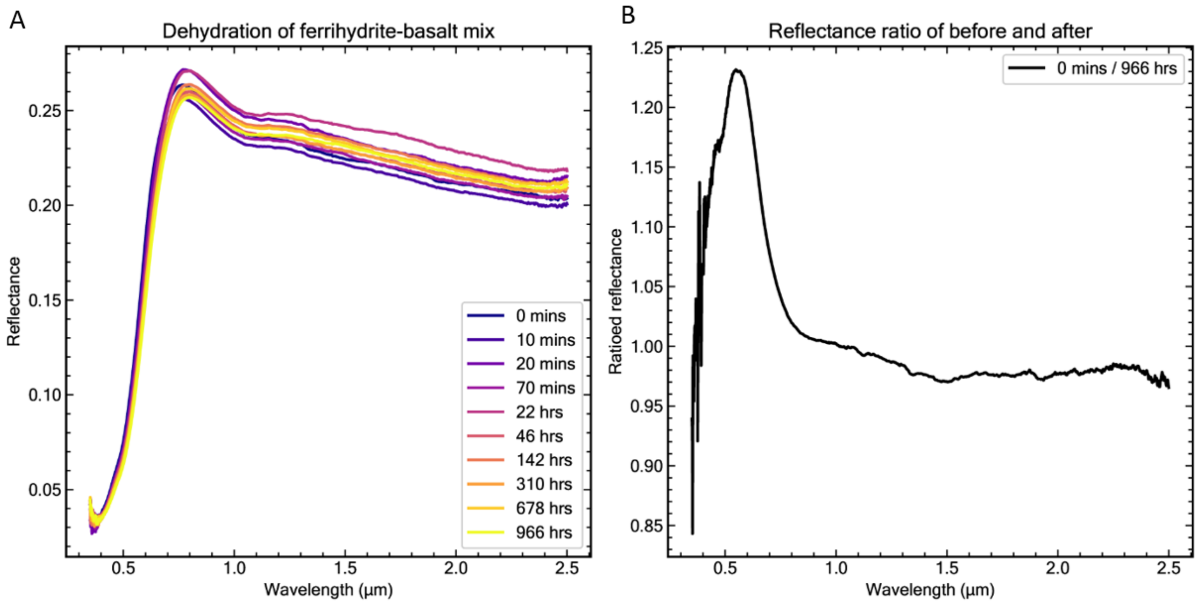

Supplementary Fig. 11. (a) Spectra of the ferrihydrite-basalt mixture under dehydrating conditions and (b) its ratioed reflectance. In (a) and (b), the ferrihydrite-basalt mixtures exhibit smaller changes after dehydration because basalt spectral features dominate this region. The loss of water may only be inferred from the changes in spectral features near the reflectance maximum at about 0.7 µm, which broadens perhaps due to the loss of crystallinity, similar to what is observed in Supplementary Fig. 12.

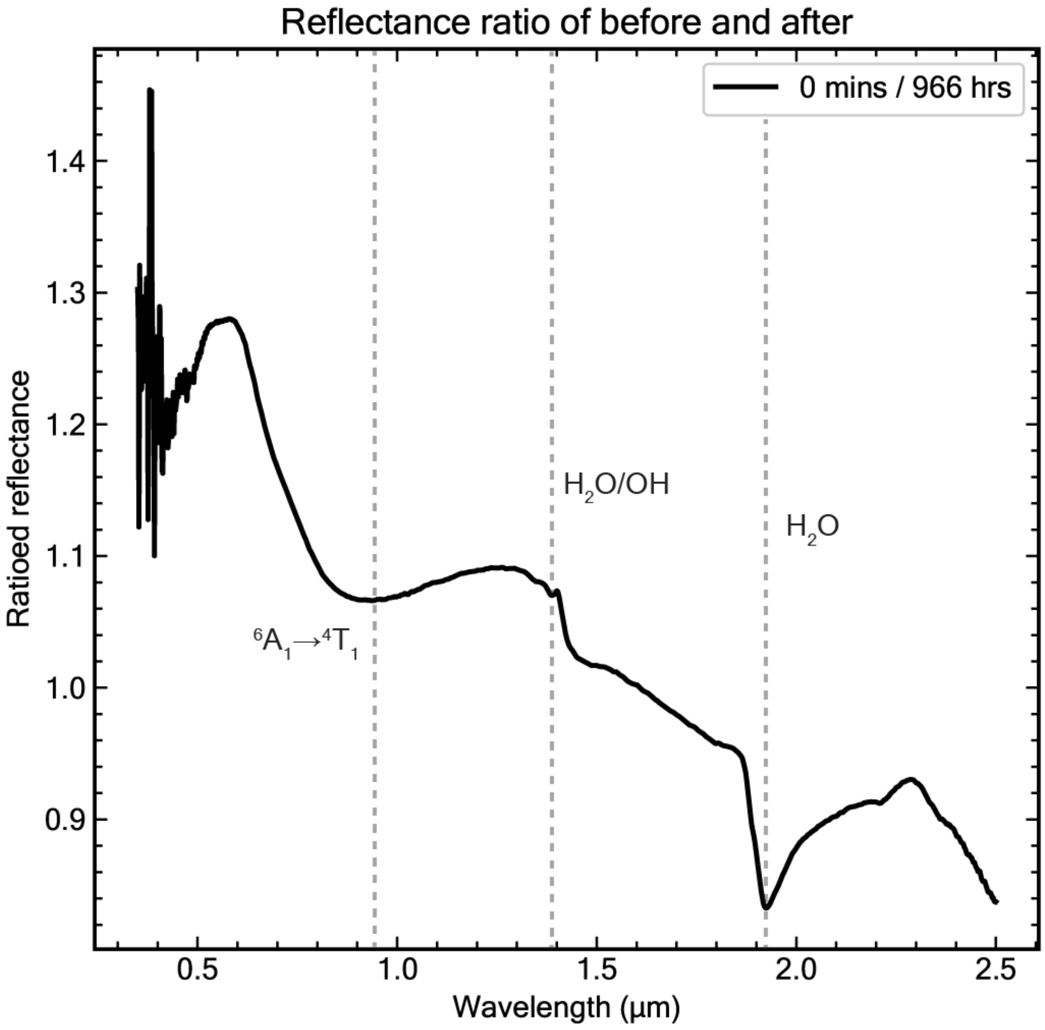

Supplementary Fig. 12. Ratioed spectra of before and after the dehydration experiment of ferrihydrite. The Fe band at ~0.95 μm is also diminished, suggesting a small structural defect due to the removal of water molecules from the crystal environment of ferrihydrite. The adjacent Fe^{3+} atoms are linked via intermediate OH/H_2O and removal of the H-bearing species may cause observed changes in the Fe band.

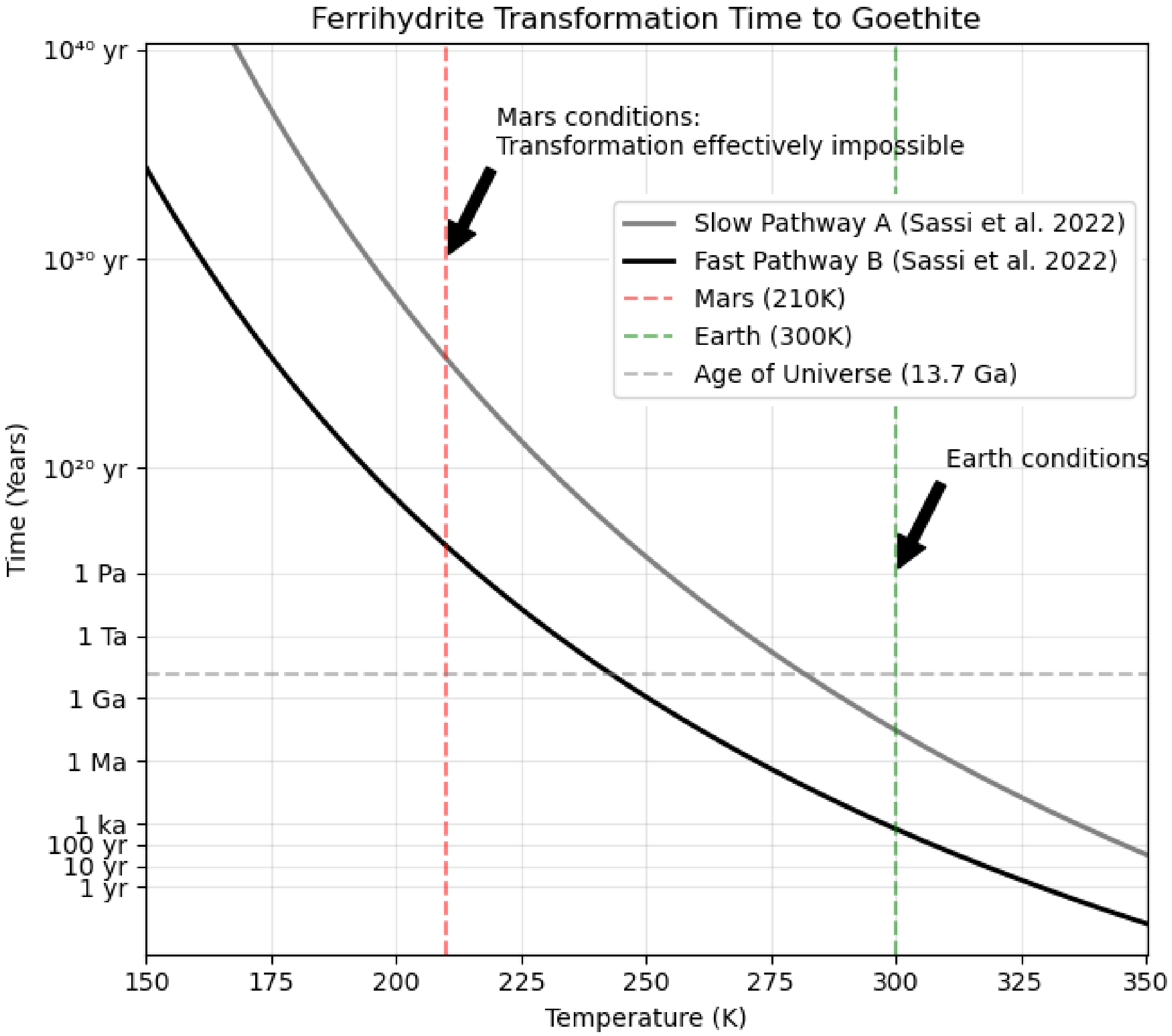

Supplementary Fig. 13. Arrhenius plot for solid-state transformation of ferrihydrite to goethite. Using activation energies from Sassi & Rosso (2022) we estimate that transformation of ferrihydrite to goethite under Martian conditions (210 K, -63° C) is highly improbable, as the required times exceed any meaningful time.

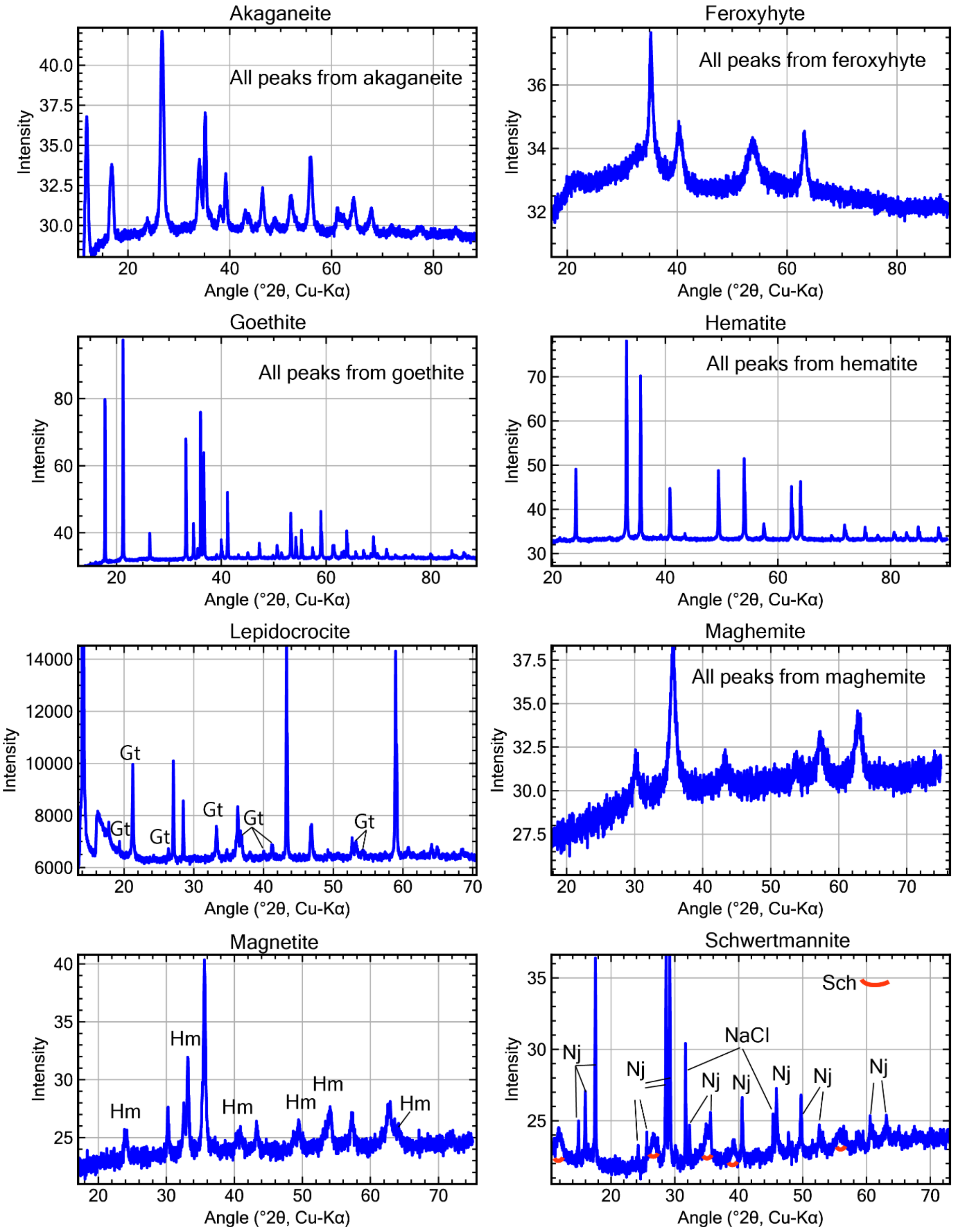

Supplementary Fig. 14. X-ray diffraction patterns of all iron oxides-hydroxides used in our study. Five out of eight samples are pure minerals. The impurities are labeled in each respective plot.

Schwertmannite's broad peaks are explicitly stated because of poor crystallinity. Gt – goethite, Hm – hematite, Nj – natrojarosite, NaCl – sodium chloride. The analysis revealed the presence of natrojarosite and NaCl impurities in the synthesized schwertmannite sample, likely due to synthesis conditions or reagent contamination. Each peak was determined using DIFFRAC.EVA software. Source data are provided as a Source Data file.

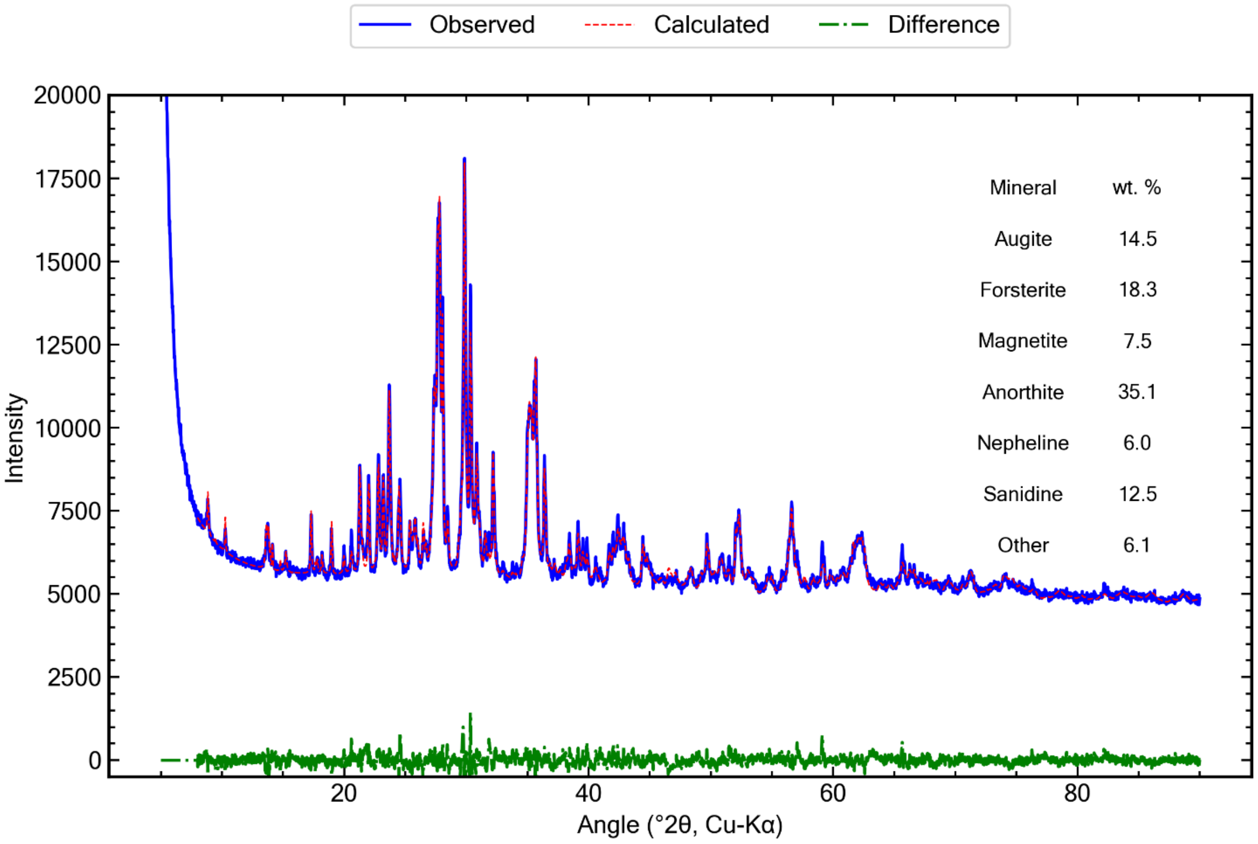

Supplementary Fig.15. Basalt powder X-ray diffraction pattern used in this study and its mineral composition. Observed (blue), calculated (red) and difference curve at the bottom (green). The high abundances of sanidine (12.5 wt%) and nepheline (6.0 wt%), along with the calculated low total SiO_2 content (40.83%), this basalt shows characteristics of an alkaline basalt rather than a tholeiitic basalt. The unusually high potassium content, evidenced by the abundant sanidine, suggests either an alkaline-rich source, magma mixing processes, or post-crystallization

alteration. The presence of nepheline confirms silica undersaturation, which is typical of alkaline basalts but contradicts tholeiitic compositions. The combination of nepheline, sanidine and anorthite points to an evolved alkaline magmatic system rather than a typical mid-ocean ridge or continental tholeiitic basalt composition. Source data are provided as a Source Data file.

References

1. Johnson, J. R. *et al.* ChemCam passive reflectance spectroscopy of surface materials at the Curiosity landing site, Mars. *Icarus* **249**, 74–92 (2015).
2. Rampe, E. B., Morris, R. V, Archer Jr, P. D., Agresti, D. G. & Ming, D. W. Recognizing sulfate and phosphate complexes chemisorbed onto nanophase weathering products on Mars using in-situ and remote observationsk. *Am. Mineral.* **101**, 678–689 (2016).
3. Poitras, J. T. *et al.* Mars analog minerals' spectral reflectance characteristics under Martian surface conditions. *Icarus* **306**, 50–73 (2018).
4. Cloutis, E. A. *et al.* Spectral reflectance properties of minerals exposed to simulated Mars surface conditions. *Icarus* **195**, 140–168 (2008).
5. Das, S., Hendry, M. J. & Essilfie-Dughan, J. Transformation of Two-Line Ferrihydrite to Goethite and Hematite as a Function of pH and Temperature. *Environ. Sci. Technol.* **45**, 268–275 (2011).
6. Das, S., Hendry, M. J. & Essilfie-Dughan, J. Transformation of Two-Line Ferrihydrite to Goethite and Hematite as a Function of pH and Temperature. *Environ. Sci. Technol.* **45**, 268–275 (2011).
7. Wang, X. *et al.* Effect of Ferrihydrite Crystallite Size on Phosphate Adsorption Reactivity. *Environ. Sci. Technol.* **47**, 10322–10331 (2013).
8. Bishop, J. L. Visible and Near-Infrared Reflectance Spectroscopy. in *Remote Compositional Analysis* (eds. Bell III, J. F., Bishop, J. L. & Moersch, J. E.) 68–101 (Cambridge University Press,

2019). doi:10.1017/9781316888872.006.

9. Sklute, E. C. *et al.* Spectral and morphological characteristics of synthetic nanophase iron (oxyhydr)oxides. *Phys. Chem. Miner.* **45**, 1–26 (2018).

10. Sassi, M. & Rosso, K. M. Ab Initio Evaluation of Solid-State Transformation Pathways from Ferrihydrite to Goethite. *ACS Earth Sp. Chem.* **6**, 800–809 (2022).
